# Uncommon Variants in *FLG2* and *NOD2* Are Associated with Atopic Dermatitis in the Ethiopian Population

**DOI:** 10.1016/j.xjidi.2024.100284

**Published:** 2024-04-29

**Authors:** Sailan Wang, Julia K. Elmgren, Jesper Eisfeldt, Samina Asad, Marlene Ek, Kassahun Bilcha, Annisa Befekadu, Carl-Fredrik Wahlgren, Magnus Nordenskjöld, Fulya Taylan, Isabel Tapia-Paez, Maria Bradley

**Affiliations:** 1 Division of Dermatology and Venereology, Department of Medicine, Solna, Karolinska Institutet, Karolinska University Hospital, Stockholm, Sweden; 2 Department of Molecular Medicine and Surgery, Karolinska Institutet, Stockholm, Sweden; 3 Department of Clinical Genetics and Genomics, Karolinska University Hospital, Stockholm, Sweden; 4 Department of Dermatovenereology, Faculty of Medicine, Gondar University, Gondar, Ethiopia; 5 U.S. Dermatology Partners, Dulles, Virginia, USA

**Keywords:** Atopic dermatitis, FLG2, Genetic association, NOD2, Whole-genome sequencing

## Abstract

Loss-of-function variants in the *FLG* gene have been identified as the strongest cause of susceptibility to atopic dermatitis (AD) in Europeans and Asians. However, very little is known about the genetic etiology behind AD in African populations, where the prevalence of AD is notably high. We sought to investigate the genetic origins of AD by performing whole-genome sequencing in an Ethiopian family with 12 individuals and several affected in different generations. We identified 2 variants within *FLG2* (p.D13Y) and *NOD2* (p.A918S) genes cosegregating with AD in the affected individuals. Further genotyping analyses in both Ethiopian and Swedish AD cases and controls revealed a significant association with the *FLG2* variant (p.D13Y, *P* < .0013) only in the Ethiopian cohort. However, the *NOD2* variant (p.A918S) did not show any association in our Ethiopian cohort. Instead, 2 previously recognized *NOD2* variants (p.A849V, *P* < .0085 and p.G908R, *P* < .0036) were significantly associated with AD in our Ethiopian cohort. Our study indicates that the *FLG2* and *NOD2* genes might be important in the etiology of AD in Ethiopians. Additional genetic and functional studies are needed to confirm the role of these genes and the associated variants into the development of AD.

## Introduction

Atopic dermatitis (AD) is the most common chronic inflammatory skin disorder and is characterized by T helper 2 cell–mediated immune response and epidermal dysfunction. The current estimated prevalence of AD is approximately 2–10% in adults and 15–30% in children (Weidinger et al, 2018). The prevalence of AD varies among populations; it is slightly higher in African American children (19.3%) than in European American children (16.1%) (Brunner and Guttman-Yassky, 2019). In Ethiopia, the prevalence of AD has been estimated to be as high as 19% in Addis Ababa and around 1–2% in Southern Ethiopia (Ait-Khaled et al, 2007).

A significant contributing factor to AD is the family history; it is estimated that if 1 parent is affected, the risk in the child increases by threefold, and if both parents are affected, the risk increases to fivefold (Weidinger et al, 2018). Today, loss-of-function variants in the *FLG* gene are the most recognized susceptibility factor for AD, increasing the risk of developing AD by 3–5 times (Laughter et al, 2021). The *FLG* gene encodes the epidermal protein FLG, a major structural protein of the stratum corneum, essential for the epidermal barrier and for maintaining hydration (Sandilands et al, 2009). Notably, the *FLG*-null variants are particularly prevalent in European and Asian populations (Smith et al., 2006), whereas the absence of the common *FLG-*null variants was indicated among AD cases in the African population, in particular, in Ethiopians (Polcari et al, 2014; Winge et al, 2011).

Beyond *FLG*, genome association studies have identified additional loci associated with AD, encompassing genes implicated in immune regulation, epidermal barrier maintenance, tissue response, and environmental sensing (Tsakok et al, 2019). However, the cumulative impact of these genes accounts for only approximately 20% of the total heritability of AD, underscoring the complexity of its genetic underpinnings.

## Results

### Variants in *FLG2* and *NOD2* genes cosegregate with AD in affected individuals of an Ethiopian AD family

**Figure 1:**
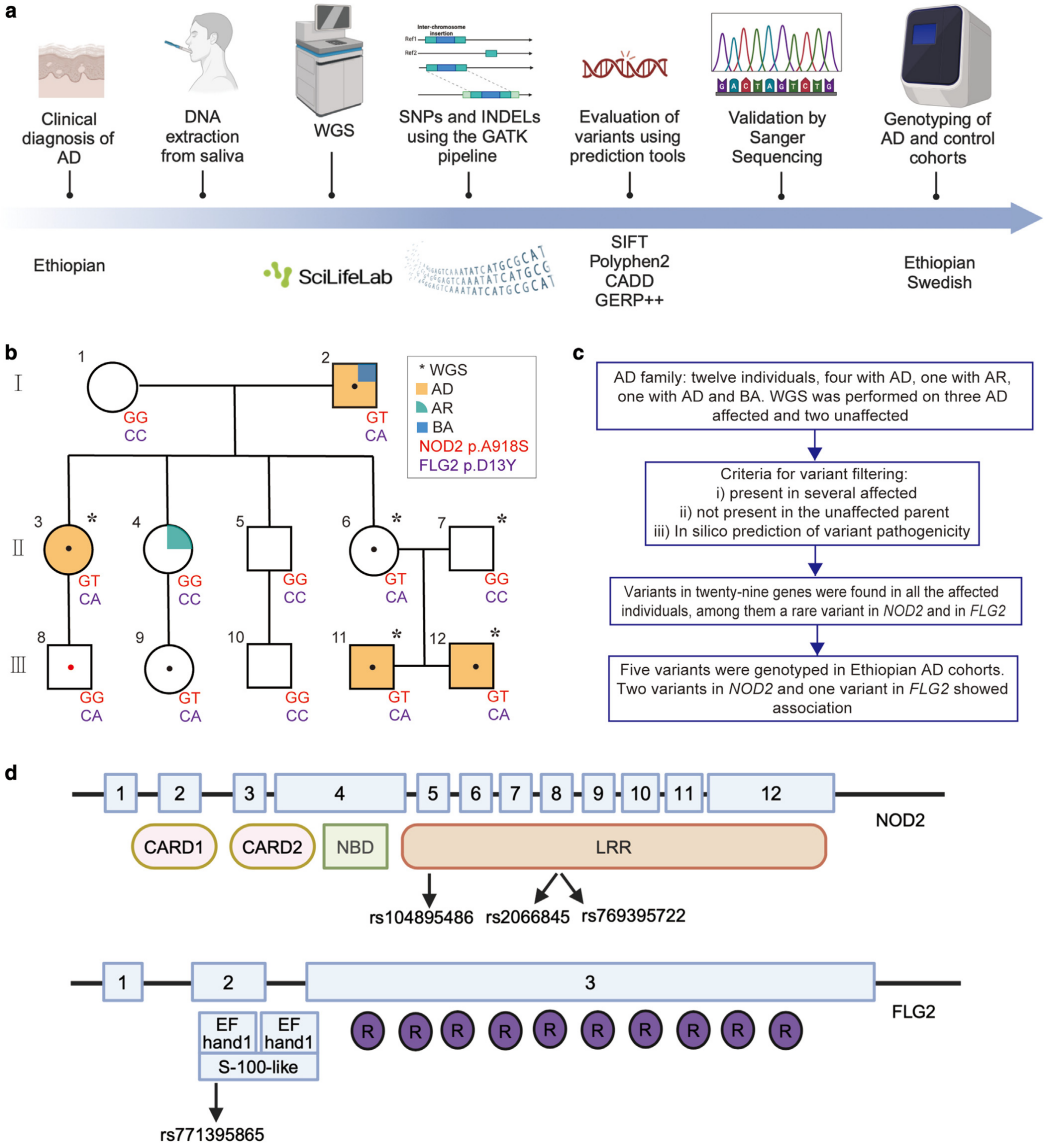
**WGS revealed rare variants in the *NOD2* and the *FLG2* gene in Ethiopian patients with AD.** (**a**) Flowchart of the pipeline involved in generating and verifying the WGS results. DNA was sequenced using the Illumina platform. The GATK pipeline was applied to identify SNPs and insertions/deletions. The prediction tools were used to assess the potential impact of variants on protein function, conservation, and pathogenicity. Sanger sequencing was used for validating nucleotide changes. Genotyping was applied in both Ethiopian and Swedish case–control cohorts. SIFT denotes Sorting Intolerant from Tolerant, PolyPhen-2_pred denotes Polymorphism Phenotyping v2 Predication, CADD denotes Combined Annotation Dependent Depletion, and GERP++ denotes Genomic Evolutionary Rate Profiling. This figure was created with BioRender.com. (**b**) Pedigree chart from the Ethiopian family studied. Basic annotations are as follows: circles and squares denote females and males, respectively. Orange-, green-, and blue-filled shapes mean affected by AD, allergic rhinitis, and bronchial asthma, respectively. WGS was performed for the samples marked with asterisks. A black dot represents a carrier of both heterozygous variants: *NOD2* p.A918S(GT) and *FLG2* p.D13Y(CA); a red dot represents a carrier of the *FLG2* p.D13Y variant. (**c**) Filtering strategy for genetic variants found in the Ethiopian family. (**d**) Location of the variants in NOD2 and FLG2. R denotes repeat. Figure 1a and d was created with BioRender.com. AR, allergic rhinitis; AD, atopic dermatitis; BA, bronchial asthma; GATK, Genome Analysis Toolkit; LRR, leucine-rich repeat; NBD, nucleotide binding domain; WGS, whole-genome sequencing.

**Figure 2:**
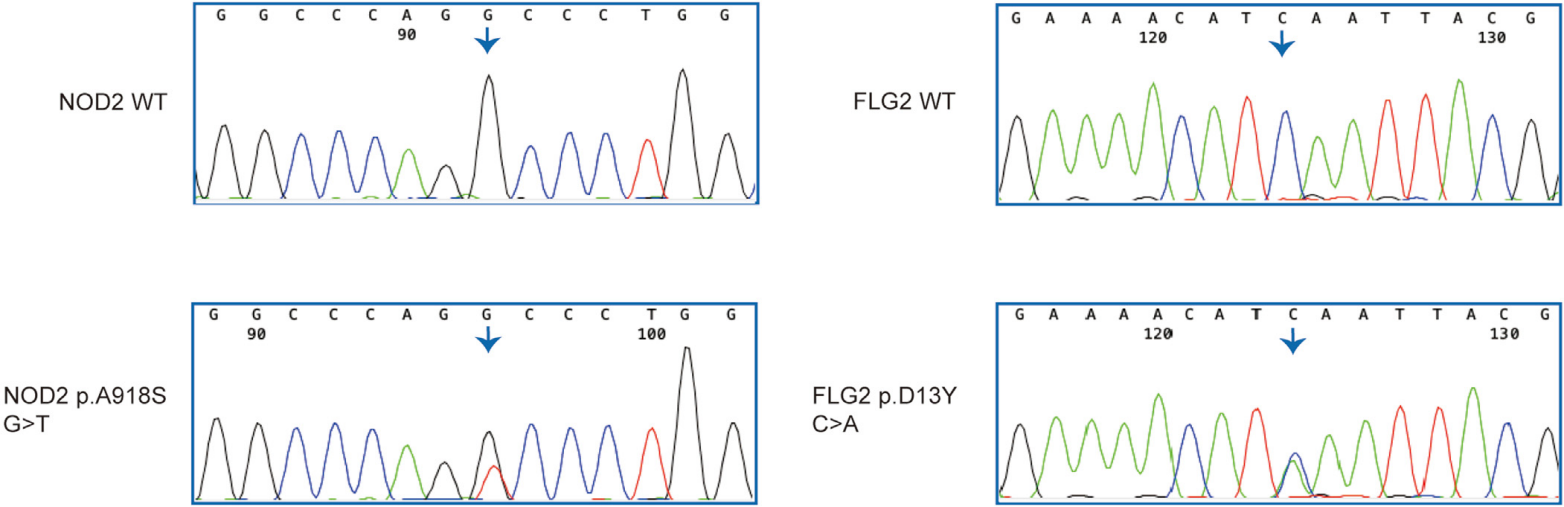
**Variant validation through Sanger sequencing.** Sanger sequencing results show the 2 missense variants p.A918S in *NOD2* and p.D13Y in *FLG2*. A denotes alanine, S denotes serine, and D denotes aspartic acid. WT, wild type.

**Figure 3:**
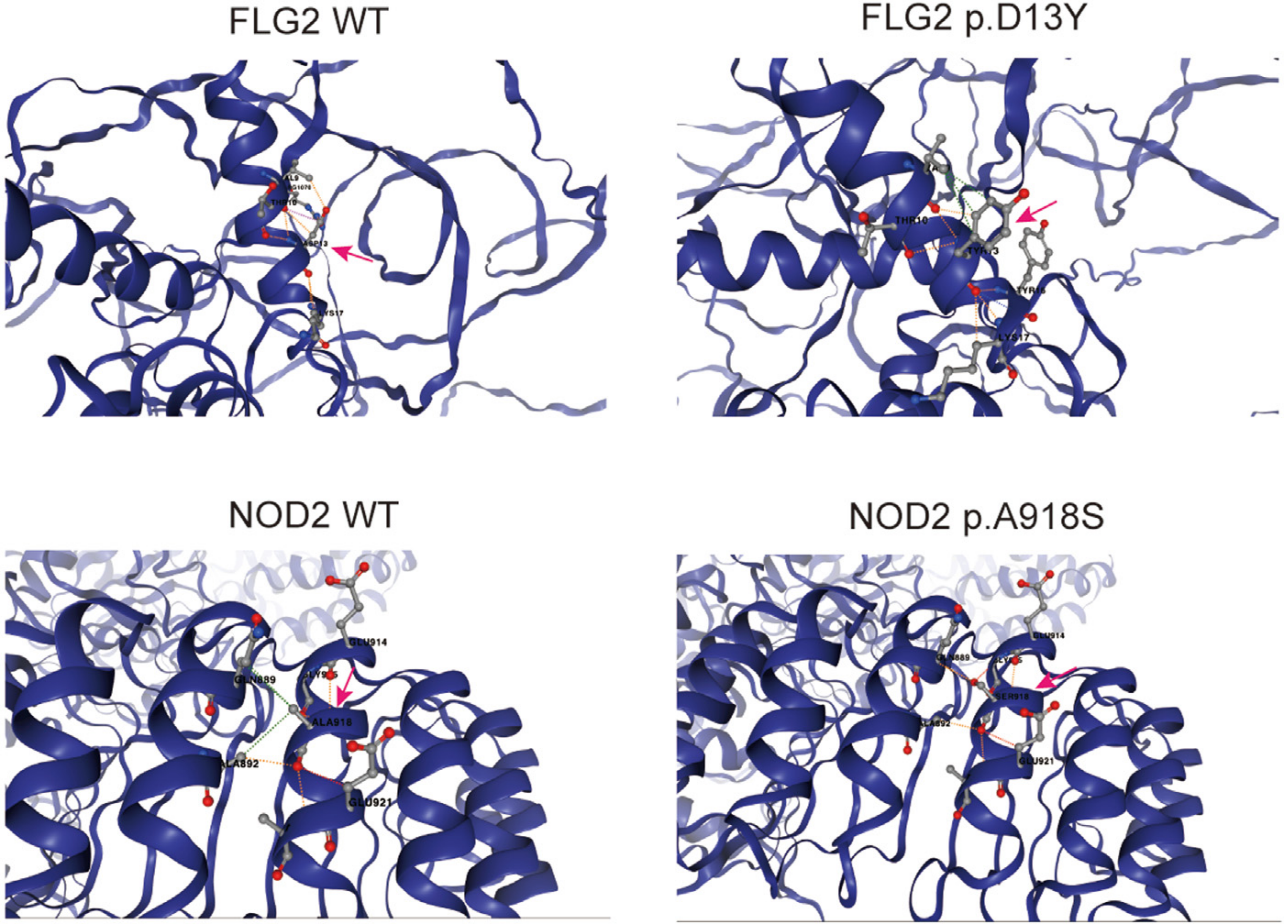
**Protein structural analysis of the genetic variants.** The analyses were performed using the DynaMut2 server, which assesses the effects of missense variants on protein stability and flexibility. The dynamut2 analysis revealed that the missense variants of *NOD2* p.A918S and *FLG2* p.D13Y could cause destabilization, and the ΔΔG stability was brought down to −0.45 kcal/mol and −0.03 kcal/mol, respectively. Amino acid changes are highlighted with arrows.

To investigate the potential susceptibility genes involved in the pathogenesis of AD in Ethiopians, we performed whole-genome sequencing (WGS) in 3 affected and 2 unaffected individuals of a 3 generation Ethiopian AD family with 12 individuals (Figure 1a and b). As described in flowchart in Figure 1c, we looked for potentially pathogenic variants that were shared among the affected individuals and not present in the unrelated family member (individual 7 in Figure 1b). Using this strategy, we identified variants in 29 genes (Table 1). Among the variants, 2 rare deleterious missense variants rs771395865 (p.D13Y; Genome Aggregation Database, version 4.0: 0.0001869 in African/African American and Combined Annotation Dependent Depletion: 22.9) in *FLG2* and rs769395722 (p.A918S; Genome Aggregation Database, version 4.0: 0.00009342 in African/African American and Combined Annotation Dependent Depletion: 25.8) were found in the *NOD2* in the 4 sequenced affected individuals, indicating cosegregation of these variants with AD (Figure 1b and Table 2). Both variants were validated using Sanger sequencing in the family (Figure 2). Protein modeling of these missense variants using DynaMut2 predicted the destabilization of the proteins (Figure 3).

**Table 1:** Genes Identified by WGS in Ethiopian Patients with AD

Gene	Name	HGNC ID	Location	Position in hg19	Start	End	Nucleotide Change
*ACSM2A*	Acyl-CoA synthetase medium chain family member 2A	HGNC:32017	16p12.3	Chr 16	20497955	20497956	C>T
*ARHGAP42*	Rho GTPase activating protein 42	HGNC:26545	11q22.1	Chr 11	100849773	100849774	C>G
*ASB6*	Ankyrin repeat and SOCS box containing 6	HGNC:17181	9q34.11	Chr 9	132401492	132401493	C>T
*BCAR1*	BCAR1 scaffold protein, Cas family member	HGNC:971	16q23.1	Chr 16	75270853	75270854	G>C
*CACNG1*	Calcium voltage-gated channel auxiliary subunit gamma 1	HGNC:1405	17q24.2	Chr 17	65040953	65040954	C>A
*CCNF*	Cyclin F	HGNC:1591	16p13.3	Chr 16	2493790	2493791	G>A
*CCNT2*	Cyclin T2	HGNC:1600	2q21.3	Chr 2	135712148	135712149	G>T
*CDH1*	Cadherin 1	HGNC:1748	16q22.1	Chr 16	68845718	68845719	A>G
*CDH1*	Cadherin 1	HGNC:1748	16q22.1	Chr 16	68849661	68849662	C>T
*DHRS3*	Dehydrogenase/reductase 3	HGNC:17693	1p36.21	Chr 1	12640561	12640562	C>T
*ERCC6L2*	ERCC excision repair 6 like 2	HGNC:26922	9q22.32	Chr 9	98740465	98740466	T>A
*FLG2*	FLG2	HGNC:33276	1q21.3	Chr 1	152331323	152331324	C>A
*GH2*	Growth hormone 2	HGNC:4262	17q23.3	Chr 17	61958786	61958787	G>A
*GRK6*	G protein-coupled receptor kinase 6	HGNC:4545	5q35.3	Chr 5	176862076	176862077	G>A
*HSPB2*	Heat shock protein family B (small) member 2	HGNC:5247	11q23.1	Chr 11	111783566	111783567	C>T
*KANK3*	KN motif and ankyrin repeat domains 3	HGNC:24796	19p13.2	Chr 19	8400435	8400436	C>T
*LOC389831*	—	—	—	Chr 7: GL000195.1	44731	44732	C>A
*LOC389831*	—	—	—	Chr 7: GL000195.1	44734	44735	G>A
*LOC389831*	—	—	—	Chr 7: GL000195.1	44904	44905	C>CCT
*LOC389831*	—	—	—	Chr 7: GL000195.1	44922	44923	C>T
*LOC389831*	—	—	—	Chr 7: GL000195.1	48990	48991	A>G
*LOC389831*	—	—	—	Chr 7: GL000195.1	74183	74184	A>G
*LONP1*	Lon peptidase 1, mitochondrial	HGNC:9479	19p13.2	Chr 19	5719787	5719788	T>C
*NOD2*	Nucleotide binding oligomerization domain containing 2	HGNC:5331	16q12.1	Chr 16	50756569	50756570	G>T
*PCM1*	pericentriolar material 1	HGNC:8727	8p22	Chr 8	17823867	17823868	C>T
*PRSS36*	Serine protease 36	HGNC:26906	16p11.2	Chr 16	31153889	31153890	C>T
*RPGRIP1L*	RPGRIP1 like	HGNC:29168	16q12.2	Chr 16	53644903	53644904	G>A
*SLC44A2*	Solute carrier family 44 member 2 (CTL2 blood group)	HGNC:17292	19p13.2	Chr 19	10745467	10745468	G>A
*SLC5A2*	Solute carrier family 5 member 2	HGNC:11037	16p11.2	Chr 16	31500220	31500221	T>A
*SWI5*	SWI5 homologous recombination repair protein	HGNC:31412	9q34.11	Chr 9	131050953	131050954	A>T
*THAP7*	THAP domain containing 7	HGNC:23190	22q11.21	Chr 22	21354437	21354438	G>A
*TMEM94*	Transmembrane protein 94	HGNC:28983	17q25.1	Chr 17	73487781	73487782	G>A
*TSEN54*	tRNA splicing endonuclease subunit 54	HGNC:27561	17q25.1	Chr 17	73519814	73519815	G>A
*TXNRD2*	Thioredoxin reductase 2	HGNC:18155	22q11.21	Chr 22	19906520	19906521	C>T
*ZFHX4*	Zinc finger homeobox 4	HGNC:30939	8q21.13	Chr 8	77767740	77767741	G>A

Abbreviations: AD, atopic dermatitis; Chr, chromosome; HGNC, Human Gene Nomenclature Committee; ID, identification; WGS, whole-genome sequencing.

**Table 2:** Characteristics of the Variants Studied

Gene	dbSNP	Position in hg19	Nucleotide Change	Amino Acid Change	Exonic Function	CADD Score	PolyPhen-2 _pred	SIFT _pred	AlphaMissense _pred	gnomAD			ALFA		NGS
										Global	AFR		Global	AFR	
*NOD2*	rs769395722	Chr 16	G>T	p.A918S	Missense	25.8	Probably damaging	Deleterious	—	0.0000	0.0000		0.0000	0.0000	WGS
*NOD2*	rs2066845	Chr 16	G>C	p.G908R	Missense	26.3	Probably damaging	Deleterious	Pathogenic	0.01288	0.0025		0.0142	0.0034	WES
*NOD2*	rs104895486	Chr 16	C>T	p.A849V	Missense	32	—	Deleterious	Pathogenic	0.0000	0.0000		0.0000	0.0000	WES
*FLG2*	rs771395865	Chr 1	C>A	p.D13Y	Missense	22.9	Probably damaging	Deleterious	Pathogenic	0.0002	0.0002		0.0000	0.0000	WGS

Abbreviations: ALFA, Allele Frequency Aggregator; CADD, Combined Annotation Dependent Depletion; Chr, chromosome; dbSNP, SNP Database; gnomAD, Genome Aggregation Database; NGS, next-generation sequencing; PolyPhen-2_pred, Polymorphism Phenotyping v2 Predication; SIFT_pred, Sorting Intolerant from Tolerant Predication; WES, whole-exome sequencing; WGS, whole-genome sequencing.

A CADD score of 30 is predicted to be the 0.1% most deleterious possible substitution in the human genome. PolyPhen-2 and SIFT are computational algorithms commonly used to predict whether or not a specific amino acid substitution in a protein, caused by a genetic variant, is likely to be damaging to protein function. AlphaMissense predictions may illuminate the molecular effects of missense variants, identify pathogenic variants, uncover novel disease-causing genes, and increase diagnostic accuracy for genetic diseases (https://github.com/google- deepmind/alphamissense). AlphaMissense_pred is a deep learning model based on the protein structure prediction tool AlphaFold2. AFR denotes African population.

### Association of *FLG2* and *NOD2* variants with AD in an Ethiopia cohort

To further investigate the association of the variants rs771395865 (p.D13Y) in *FLG2* and rs769395722 (p.A918S) in *NOD2* identified in the family, we genotyped Ethiopian and Swedish AD cases and controls. The Ethiopian cohort consisted of 189 cases and 203 controls, whereas the Swedish cohort included 300 cases and 2000 controls. In addition to investigating the identified variants in the Ethiopian family, we also genotyped the *NOD2* variants (p.A849V and p.G908R), observed previously in a study where whole-exome sequencing was performed in 22 Ethiopian patients with AD (Taylan et al, 2015). The frequencies of these variants, globally and in Africans, were extracted from the Genome Aggregation Database and Allele Frequency Aggregator databases and are described in Table 2.

The *FLG2* p.D13Y variant (*P* < .0003) as well as the *NOD2* p.A849V (*P* < .0085) and p.G908R (*P* < .0036) variants were found to be statistically significant in the Ethiopian AD cases compared with those in Ethiopian healthy controls. However, these variants were not found to be significant in the Swedish AD case–control comparison (Table 3), suggesting that their importance in susceptibility to AD is limited to certain populations, including Ethiopians. The *NOD2* p.A918S variant (*P* < .469) was not found to be associated with either of the populations (Table 3).

**Table 3:** Genotyping of Selected Variants in Ethiopian and Swedish Case–Control Cohorts

Gene	SNP	Minor Allele	Genotype	Ethiopia		OR	*P*-Value	MAF			Sweden		*P*-Value
				Controls	AD			Controls	AD		Controls^1^	AD	
*NOD2*	rs769395722	T	CC	197	153	0.67	.469	0.0148	0.0219		2000	300	—
			CT	6	7	3.5					0	0	
*NOD2*	rs2066845	C	GG	181	131	0.28	.0036	0.0186	0.0604		1994	297	.3123
			CT	7	18	3.55					6	2	
*NOD2*	rs104895486	T	GG	202	143	0	.0085	0	0,0169		2000	300	.2728
			GT	0	5	14.13					0	0	
*FLG2*	rs771395865	T	CC	186	151	0.32	.0003	0.0373	0.1005		2000	300	.2728
			CT	15	38	3.12					0	0	

Abbreviations: AD, atopic dermatitis; MAF, minor allele frequency.

1 Data were extracted from https://swefreq.nbis.se/.

### Expression of FLG2 and NOD2 in skin biopsies of Ethiopian patients carrying the associated variants

**Figure 4:**
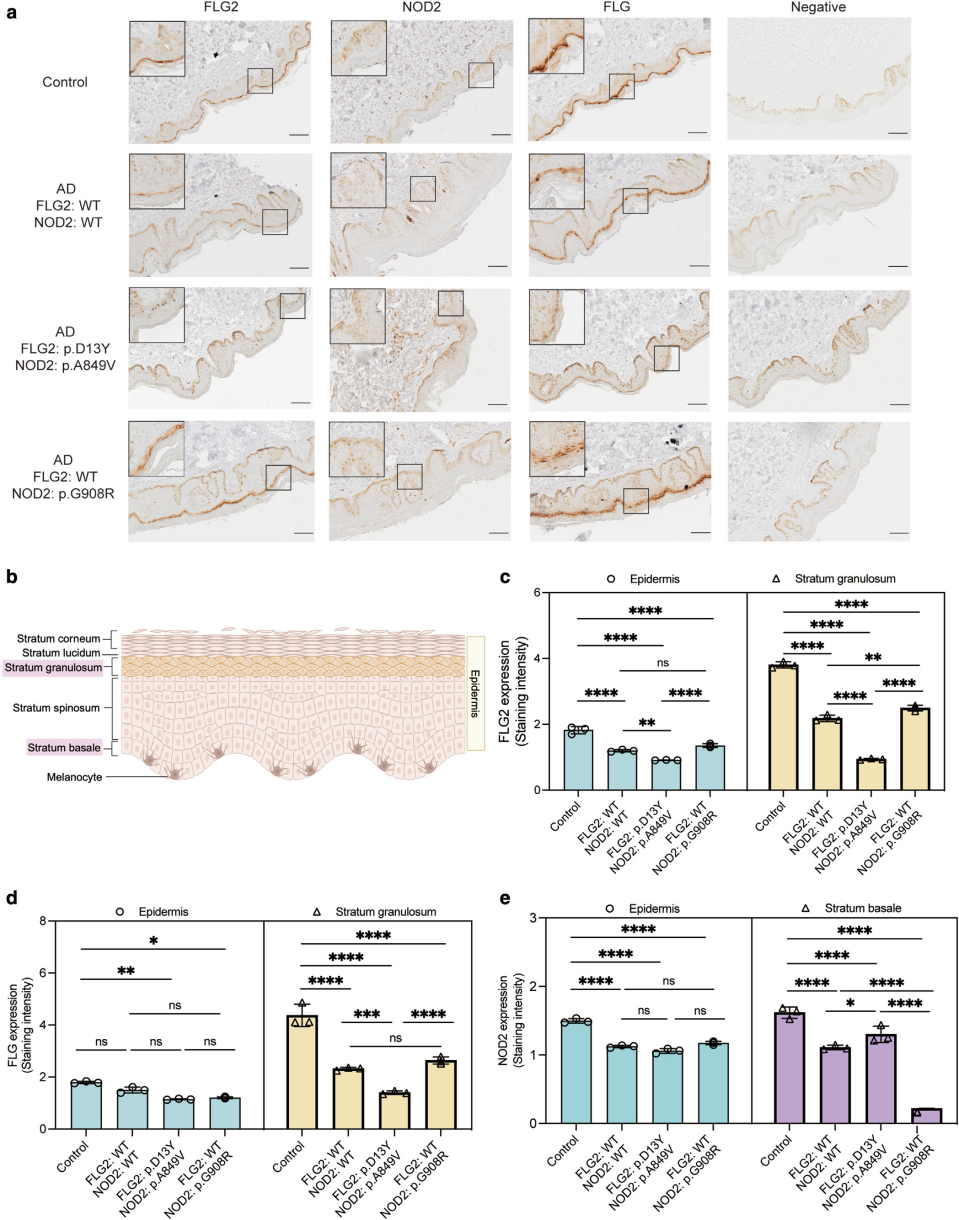
**Immunohistochemical staining for FLG2, NOD2*,* and FLG in skin biopsies from 3 Ethiopian patients and a healthy control.** (**a**) The first patient was a noncarrier of the variants, the second was a carrier of the *FLG2* p.D13Y and *NOD2* p.A849V variants, and the third was a carrier of the *NOD2* p.G908R variant only. Brown 3,3′-diaminobenzidine staining revealed variations in epidermal cell expression levels. Bars = 100 μm. (**b**) Structure and skin cell composition of the epidermis. Epidermis analysis encompassed the following layers: the stratum basale, stratum spinosum, stratum granulosum, stratum lucidum, and stratum corneum; the assessment for FLG2 specifically focused on the stratum granulosum (highlighted in pink), whereas stratum basale (highlighted in pink) is for NOD2. This figure was adapted from Skin Cancer Progression by BioRender.com (2020). Retrieved from https://app.biorender.com/biorender-templates. (**c–e**) Staining intensity quantification was conducted using ImageJ software (National Institutes of Health, Bethesda, MD, https://imagej.nih.gov/ij/). Quantification of FLG2 and FLG expression was performed in the entire epidermis and stratum granulosum, and that of NOD2 was performed in the entire epidermis and stratum basale. To ensure data robustness, all comparisons were normalized against negative controls. Error bars in figures represent mean ± SEM. Dots represent the quantification of each area for each slide. Statistical analysis was performed using ordinary 1-way ANOVA with Tukey’s multiple comparison test in GraphPad Prism, version 9.1.2 (Dotmatics, Boston, MA). Significance levels are denoted as follows: ∗*P* < .05, ∗∗*P* < .01, ∗∗∗*P* < .001, and ∗∗∗∗*P* < .0001.

To further explore the effects of the variants found in *FLG2* and *NOD2* in the protein expression, we obtained skin biopsies from 4 Ethiopian individuals: a healthy control, a patient with AD not carrying any of the studied variants, a patient with AD carrying *NOD2* p.G908R, and a patient with AD carrying the *FLG2* p.D13Y and *NOD2* p.A849V variants (Figure 4a). In addition, AlphaMissense, a prediction tool for pathogenicity of missense variants (Cheng et al, 2023), predicted the variants mentioned earlier—*NOD2* p.A849V, p.G908R, and *FLG2* p.D13Y—to be pathogenic (Table 4).

**Table 4:** Pathogenicity of Missense Variants Associated with Patients with AD in Ethiopian Patients Using AlphaMissense Predictions

Gene	Protein Variant	Pathogenicity	Class
*NOD2*	p.A849V	0.7125	Pathogenic
*NOD2*	p.G908R	0.85	Pathogenic
*FLG2*	p.D13Y	0.8766	Pathogenic

Abbreviation: AD, atopic dermatitis.

AlphaMissense predictions is a deep learning model that builds on the protein structure prediction tool AlphaFold2, which identifies the variants to be benign (0–0.34), ambiguous (0.34–0.564), and pathogenic (0.564–1.0).

Interestingly, immunostaining of the skin tissues showed that the expression of FLG2 was reduced in the presence of the *FLG2* p.D13Y variant, which also influenced the expression of FLG in the stratum granulosum (Figure 4b–d). The expression of NOD2 was lower in the presence of the p.G908R variant, but no difference was observed for the p.A849V variant compared with the epidermis and the stratum basale from lesional skin of a noncarrier patient with AD (Figure 4e).

## Discussion

Genetics plays an important role in the risk of developing AD as well as in predicting its severity. It has been observed that the prevalence of AD varies greatly between diverse ethnic groups in countries with multiethnic populations, and the reasons for the geographical and ethnic differences are not well-understood (Kaufman et al, 2018). Our findings provide further support for the idea that European and Ethiopian populations show differing genetic backgrounds in AD etiology. Furthermore, we propose that the *FLG2* and *NOD2* genes may contribute to the development of AD specifically in the Ethiopian population. This underlines the importance of including a diverse genetic background when exploring the pathogenesis of AD as well as when designing clinical trials.

The epidermal differentiation complex region on chromosome 1 contains several important genes that contribute to the structural and functional integrity of the epidermal barrier (Hoffjan and Stemmler, 2007). *FLG* and *FLG2*, which are located next to each other, are in the epidermal differentiation complex region. *FLG* is strongly associated with AD in Europeans, whereas variants in *FLG2*, which is structurally very similar to *FLG*, have previously been described in African American patients with AD and linked to more persistent forms of AD (Margolis et al, 2014). Decreased expression of *FLG2* has been associated with a thinner epidermis (Berna et al, 2022). Interestingly, when we further investigated the variants found in the Ethiopian family at the population level, we discovered that the *FLG2* p.D13Y variant was associated with AD in the Ethiopian population cohort.

*NOD2* encodes a cytosolic receptor involved in bacteria recognition by antigen-presenting cells and stimulates immune response (Negroni et al, 2018). Variants in *NOD2* are associated with Crohn’s disease (Hugot et al, 2001) and have also been shown to possibly confer susceptibility to atopic disorders in 2 German cohorts (Kabesch et al, 2003; Weidinger et al, 2005).

Even though we did not observe statistical significance in the Ethiopian AD cohort, and none of the family members reported to be affected by Crohn’s disease, the *NOD2* p.A918S variant found in the Ethiopian family affects the leucine-rich repeat domain, important for bacterial recognition, thereby potentially playing a critical role in innate immunity (Figure 1d).

Our results prompted us to investigate other variants in *NOD2* previously suggested to be associated with AD, such as *NOD2* p.A849V and p.G908R (Kabesch et al, 2003; Weidinger et al, 2005). Both *NOD2* variants showed significant association in our Ethiopian case–control cohort, suggesting that both *FLG2* and *NOD2* genes may play a role in the etiology of AD in the Ethiopian population.

Furthermore, reduced expression of FLG, FLG2, and NOD2 in the staining of skin biopsies from 2 Ethiopian patients with AD carrying the associated variants supports the association. Missense variants such as the ones associated with AD in Ethiopians presented in this study may affect protein folding, potentially influencing protein expression. Even though *NOD2* p.A849V, p.G908R, and *FLG2* p.D13Y variants were predicted to be pathogenic using in silico tools such as AlphaMissense, in vitro assays are needed to confirm the functional consequences of these variants.

Our study is limited by the cohort sizes. Because the identified variants were rare in databases (minor allele frequency < 0.01), increasing the number of participants would have improved the power of our study. However, our study suggests that the identified variants in *NOD2* and *FLG2* associated with AD may be specific to Ethiopians. The reason why loss-of-function variants in *FLG* are not associated with AD among Ethiopians remains unknown (Fernandez et al., 2017). *NOD2* has been related to atopic diseases because it also seems to affect T helper 2 pathways, consistent with observations in AD (Kabesch et al, 2003). To fully understand the effects of the identified variants, additional studies are required for functional characterization.

## Materials and Methods

### Saliva collection and genomic DNA extraction

In both Ethiopia and Sweden, the diagnosis of AD was confirmed by dermatologists, using clinical examination, a standardized questionnaire regarding other atopic manifestations, and the UK Working Party’s diagnostic criteria (Winge et al, 2011). To gather comprehensive information regarding AD severity and associated phenotypes such as allergies, questionnaires were administered to all patients and controls (Asad et al, 2019; Taylan et al, 2015; Winge et al, 2011). Saliva samples were collected using the Oragene-Discover (OGR-600) kit from DNAGenotek. In this study, we received saliva samples from a single family residing in Ethiopia, comprising a total of 12 individuals, including patients diagnosed with AD (n = 4) (Table 5). Ethiopian individuals diagnosed with AD (n = 189) and a control group (n = 203), consisting of subjects without any history of AD, dry skin, or atopic manifestations, were recruited at the dermatology department of Black Lion University Hospital in Addis Ababa and the University of Gondar (Gondar, Ethiopia). For comparison, saliva samples were collected from patients with AD at Karolinska University Hospital (Stockholm, Sweden) (n = 300). Data for Swedish healthy controls (n = 2000) were obtained from the SweGen database (Ameur et al, 2017).

**Table 5:** Demographic and Clinical Characteristics of Individuals in the Ethiopian family

Sample	Age, y	Sex	AD SCORAD	Atopic Manifestations
1	70	F	n.a.	n.a.
2	72	M	18	Bronchial asthma
3	35	F	54	n.a.
4	40	F	n.a.	Allergic rhinitis
5	32	M	n.a.	n.a.
6	30	F	n.a.	n.a.
7	46	M	n.a.	n.a.
8	10	M	n.a.	n.a.
9	7	F	n.a.	n.a.
10	5	M	n.a.	n.a.
11	6	M	9	n.a.
12	3	M	27	n.a.

Abbreviations: AD, atopic dermatitis; F, female; M, male; n.a., not applicable; SCORAD, SCORing Atopic Dermatitis.

Genomic DNA was extracted with prepIT.L2P reagents (DNAGenotek), following the manufacturer’s instructions. Briefly, the prepIT.L2P reagent was added to the saliva samples, and the tubes were then centrifuged. The DNA pellet was washed, centrifuged, and air dried. Then, the DNA pellet was resuspended in TE buffer, and DNA quantification was performed using a Nanodrop1000 spectrophotometer.

### WGS of an Ethiopian family, including patients with AD

WGS was performed on 3 patients with AD and 2 healthy individuals from an Ethiopian family using high-yield and high-quality DNA samples. The DNA of the male in the first generation failed to meet the WGS quality control criteria. The sequencing was carried out at the National Genomics Infrastructure at the Science for Life Laboratory (Stockholm, Sweden). For library preparation, high-quality genomic DNA samples (300 ng) were pooled and sequenced on a 1 Illumina NovaSeq6000 S4 lane (Illumina, San Diego, CA) with 2 × 150 bp pair-end reads with DNA PCR-Free kits (350 bp insert size). The resulting data were processed, and the sequence reads were aligned to the human genome build GRCh37. SNPs and insertions/deletions were detected using the Genome Analysis Toolkit pipeline. Genetic variants were annotated using a toolbox (eg, dbSnp and SnpEff). The variant selection was based on the following criteria: (i) present in several or all affected individuals, (ii) absent in unaffected parent, and (iii) in silico prediction of pathogenicity. The impact of variants was evaluated using the variant pathogenicity classifiers, including SIFT (Sorting Intolerant From Tolerant), Polyphen2 (Polymorphism Phenotyping, version 2), Combined Annotation-Dependent Depletion, GERP++ (Genomic Evolutionary Rate Profiling), and Google DeepMind AlphaMissense (Cheng et al, 2023). Ultimately, the selected variants were manually examined in BAM (Binary Alignment Map) files using the Integrated Genomics Viewer.

### Sanger sequencing

To confirm the variants identified through WGS in the Ethiopian family, Sanger DNA sequencing was conducted using the ABI 3730 PRISM DNA Analyzer at the KIGene Core Facility. Pairs of primers were designed and picked using the Primer3web (version 4.1.0). The primers used in the study are shown in Table 6.

**Table 6:** Primers Used for Genomic DNA Sanger Sequencing

Gene	SNP	Forward	Reverse
*NOD2*	rs769395722/rs2066845	GCATTGAGGCTGGGGAATAA	TGACGTTCTTTGCCAGCATC
*NOD2*	rs104895486	GGCTCTGTATTTGCGCGATA	AAGAAGTTCTGCCTGCATGC
*FLG2*	rs771395865	AGCTTGTACAGGACTCCAGC	AGATGACCGACCTCTTGAGA

### SNP genotyping

The SNPs were genotyped using the QuantStudio 6/7 Flex Real-Time PCR System Instrument (Life Technologies). Allele-specific Taqman MGB probes labeled with fluorescent dyes FAM and VIC (Applied Biosystems) were used, in accordance with the manufacturer’s instructions. QuantStudio Real-Time PCR Software (Applied Biosystems) was used for allelic discrimination analysis. All the probes used in this study were either predesigned or designed by Thermo Fisher Scientific. The list of TaqMan SNP Genotyping Assays used in this study is as follows: rs769395722, ANAAUA4; rs2066845, C_11717466_20; rs104895486, C_152958082_10; rs771395865, ANKCM77; and rs12568784, C_11261511_10.

### Immunohistochemistry of paraffin skin biopsies

The skin biopsies were obtained from 1 normal healthy control and lesional skin in 3 Ethiopian patients with AD. One of the patients was wild type for the variants examined in this study. Another patient was a carrier of rs771395865 (p.D13Y) in *FLG2* and rs104895486 (p.A849V) in *NOD2*, and the third patient was a carrier of rs2066845 (p.G908R) in *NOD2*. After fixation and paraffin embedding, 5-μm-thick sections were mounted onto glass slides. Heat-induced antigen retrieval using a pressure cooker was performed after deparaffinization. The slides were then blocked for 40 minutes with PBS 10% goat serum (Thermo Fisher Scientific) in 1% BSA/PBS + 0.2% Triton X-100. Slides were incubated overnight with primary antibodies FLG2 (rabbit, ab122011, Abcam, diluted 1:2000), NOD2 (mouse, ab31488, Abcam, diluted 1:1000), and FLG (mouse, ab218395, Abcam, diluted 1:1000). The sections were incubated with secondary biotinylated antibodies for 40 minutes at room temperature and avidin-biotin reagents (PK4000, VECTASTAIN ABC-HRP Kit) for 30 minutes. The slides were then rinsed in PBS with Tween and exposed to ImmPACT DAB substrate (SK-4105, Vector Laboratories) for 1.5 minutes.

### Quantification of immunohistochemistry

Three distinct areas (942.6 × 600.1 μm) for each slide were analyzed. To evaluate the specificity of the immunohistochemistry results, negative controls in immunohistochemistry were used, where the primary antibody was omitted. In addition, the goat anti-Mouse IgG secondary antibody was utilized and to identify any false-positive staining reactions.

The slides were converted to whole-slide imaging using an Olympus slide scanner. The entire set of slides was scanned utilizing the Olympus OlyVIA V3.3 software, enabling comprehensive viewing and the option to incorporate scale bars for suitable figures. Then, the average intensity of staining was quantified using ImageJ software and was normalized against negative immunohistochemistry controls. Through the selection in ImageJ Please draw the area of interest, the epidermis analysis included the stratum basale (the deepest portion of the epidermis), stratum spinosum, stratum granulosum, stratum lucidum, and stratum corneum (the most superficial portion of the epidermis); Specifically, for FLG2, the analysis incorporated the stratum granulosum, and for NOD2, the stratum basale was considered (Figure 2b). The results were expressed as a ratio, with a score of 1.0 indicating equal intensity and a score ≥ 1.0 representing stronger staining intensity.

### Statistical analysis

Mendelian inheritance and SNP frequencies were checked in all samples, including families and controls in the case–control cohort. Controls were checked for adherence to the Hardy–Weinberg equilibrium. Chi-square test was carried out to test for SNP association between AD and control cohorts, and *P* < .05 was considered significant. One-way ANOVA was used to assess statistically significant differences between the different groups for immunohistochemistry (∗*P* < .05, ∗∗*P* < .01, ∗∗∗*P* < .001, and ∗∗∗∗*P* < .0001).

## Ethics Statement

The study received approval from the Ethics Review Board of the University of Gondar (Gondar, Ethiopia) (RCS/05/433/2010) and the Regional Ethics Committee, Karolinska Institute (Stockholm, Sweden) (2010.345-31/2). All participants in this study provided both verbal and written consent, ensuring compliance with the principles of the Declaration of Helsinki.

## Data Availability Statement

All data supporting the findings of this study are presented within the main text. Additional data is available from the corresponding author upon request.

## ORCIDs

Sailan Wang: http://orcid.org/0000-00021269-0649

Julia K. Elmgren: http://orcid.org/0000-0002-0790-7612

Jesper Eisfeldt: http://orcid.org/0000-0003-3716-4917

Samina Asad: http://orcid.org/0009-0000-8506-6516

Marlene Ek: http://orcid.org/0000-0001-6611-309X

Annisa Befekadu: http://orcid.org/0000-0001-8098-5468

Carl-Fredrik Wahlgren: http://orcid.org/0000-0002-1769-1691

Magnus Nordenskjöld: http://orcid.org/0000-0002-4974-425X

Fulya Taylan: http://orcid.org/0000-0002-2907-0235

Isabel Tapia-Páez: http://orcid.org/0000-0002-0535-4233

Maria Bradley: http://orcid.org/0000-0001-7192-5041

## Conflict of Interest

The authors state no conflict of interest.
